# Unlocking farm-level antimicrobial resistance: a qualitative study of researchers’ experiences and challenges

**DOI:** 10.3389/fvets.2025.1691572

**Published:** 2025-11-07

**Authors:** Charles Adegbole, Togzhan Seilkhanova, Muhammad Rashid Bajwa, Rebecca Lee Smith

**Affiliations:** 1 Department of Pathobiology, University of Illinois, Urbana-Champaign, IL, United States; 2 Carl R. Woese Institute for Genomic Biology, University of Illinois, Urbana-Champaign, IL, United States; 3 School of Information Sciences, University of Illinois, Urbana-Champaign, IL, United States

**Keywords:** antimicrobial resistance, data privacy, farm, livestock, antibiotic resistance, drug resistance, dashboard

## Abstract

Despite the strong advocacy for innovation in livestock antimicrobial resistance research, a significant knowledge gap exists regarding the social components of livestock AMR research, especially as it concerns researchers. Prior studies have shown a negative perception among farm stakeholders toward antimicrobial resistance research on farms, reducing the effectiveness of such research. Furthermore, the encounters of researchers working on antimicrobial resistance in livestock settings remain underreported. This study aims to understand the experiences of researchers who conduct antimicrobial resistance research using livestock data to identify the social and technical barriers for researchers and highlight improvement opportunities. We used a semi-structured interview format to collect information from 48 researchers who had experience conducting antimicrobial resistance research on U.S. farms. Three themes captured scenarios contributing to the existing limitations around antimicrobial resistance data generated from farm settings: Navigating access and relationships impacts the quality of the research; despite challenges, more farm-level AMR data is needed because it includes crucial metadata; and preserving data integrity requires data sharing protocols. A fourth theme, researchers want to transform data into impact, described researchers’ common use of AMR data and their rationale for needed improved access. To achieve significant advancement in antimicrobial resistance in the near future, it is imperative to address the barriers that hinder access to and sharing of farm-level AMR data through development of policies and best practices.

## Introduction

Livestock production is a pillar of the agricultural sector, contributing significantly to the national economy in many countries. In the United States, it accounts for more than half of the value of agricultural commodities and contributes significantly to the gross domestic product (GDP). As a thriving industry, it contributes to employment, food security, and economic growth (1, 2). However, it is currently faced with many challenges, one of which is antimicrobial resistance (AMR). AMR is observed when organisms such as bacteria, fungi, parasites, or viruses evolve to be resistant against antimicrobials that are expected to kill or prevent their growth—making it a threat to farm productivity, livestock health, and public health (3). It has been highlighted as one of the biggest public health threats due to its status as a quintessential One Health challenge, given its capacity for transmission among humans, animals, and the environment. According to estimates from 204 countries, if antimicrobial resistance (AMR) remains unaddressed by 2050, it could lead to an annual livestock production loss of $575 billion in cumulative gross domestic product (GDP) (4). Both Small- and large-scale farmers are vulnerable to AMR as they often experience substantial financial losses due to a reduction in the performance of affected herds and flocks, increasing treatment costs, and decreased animal health. Resistance has become such a prevalent problem that it has been observed in almost every antimicrobial, including last-resort drugs like colistin (5).

The United States has implemented measures such as the development of the National Action Plan for Combating Antibiotic-Resistant Bacteria to address the growing problem of resistant organisms. Some key objectives in the action plan include fostering collaborations among all antimicrobial resistance stakeholders and developing innovative approaches to improve existing AMR testing and treatment methods. The plan also highlights the need for a collaborative effort from all stakeholders, including those concerned with animal, human, and environmental health (6). These recommendations are particularly useful in the livestock sector, as previous work suggests a decline in the willingness of agricultural stakeholders to contribute quality AMR data to the field. While the reasons for the trend remain ambiguous, the study carried out by Fynbo and Jensen (7) describes how the negative narratives about farmers’ role in AMR transmission influence farmers’ views on on-farm research and AMR data sharing. Additionally, the agricultural production sector is characterized by complex social dynamics, presenting researchers with the task of handling this complexity to drive innovation and develop sustainable solutions to AMR-related issues (8).

Researchers play a pivotal role in providing evidence-based insights into the mechanism of antimicrobial resistance and the factors driving its spread (9, 10). The work of researchers also drives advancement in techniques and tools used in exploring antimicrobial resistance within human, animals and the environment (11). Although researchers push innovation, studies show that their experience in the research process can largely affect the impact and quality of their work (12–14). This point is underscored by George and Scatolini (15) who surveyed 714 researchers from higher institutions in Oman. They found that several factors, including the inability of a researcher to obtain data from public or private institutions, can pose a significant obstacle and negatively affect a researcher’s productivity and output. In the case of antimicrobial resistance, obstacles in the research process culminate in gaps in our understanding and a risk of higher global prevalence.

Although significant efforts have been made to discover technological tools for AMR detection and data analysis in livestock, there is a gap in our understanding of researchers’ experiences and the challenges they encounter in the livestock AMR research process. Exploring these experiences is an essential prelude to identifying the barriers in antimicrobial resistance research and informing strategies to foster healthy collaborations among all stakeholders. This study aims to bridge the gap by using a qualitative approach to dive into the challenges and experiences of researchers who conduct AMR research on farms in the United States. These experiences can be used to develop guidelines, best practices, and policies around farm AMR research, including privacy protocols, data sharing, and collaboration agreements.

## Method

Given the exploratory nature of this study aimed at capturing the lived experiences of researchers, a qualitative method was considered necessary because it allows a thorough exploration of complex social phenomena that cannot be adequately captured with a quantitative measure. We used a thematic analysis approach to explore livestock AMR researchers’ lived experiences in the United States. This approach has been described previously (16). Briefly, a multifaceted approach was employed to recruit participants for the study. First, solicitation calls were distributed through email list serves with members interested in farm antimicrobial resistance and shared on social media platforms (LinkedIn, Mastodon, and Bluesky) to reach a wide range of professional communities. In addition, 218 relevant published studies addressing the analysis and interpretation of AMR data from farms in the United States were pulled from the PubMed database, and all author names were extracted into Excel (v2312). Requests for participation in the study, including the study objectives, were emailed to the identified participants. The following inclusion criteria had to be met to be eligible for the study: be at least 18 years old at the time of the interview and be familiar with analysis and interpretation of AMR farm data from the United States.

Data were collected by one researcher (TS) through a semi-structured interview over Zoom (v 5.17.7), a video conferencing software. The interviews were conducted between the 17th of October 2023 and ended on the 14 of February 2024 and lasted 25 to 60 min. The interviews were auto-transcribed using Zoom (v 5.17.7), and transcriptions were later revised and edited concurrently with the interview audios by both CA and TS. Afterward, the transcripts were de-identified and imported into MAXQDA (v22.8.0), a software for qualitative analysis. Inductive analysis began with the creation of a list of codes of interest (17). The investigators then familiarized themselves with the data by reading the transcripts to identify initial ideas and patterns and to generate other relevant codes. The data was then coded independently by CA and TS. New codes generated during the analysis process were reviewed by a third investigator (RS), who made the decision to include or exclude the code. Newly generated codes were then added retroactively during the analysis process. Codes were thereafter grouped into more prominent themes that reflected meaningful patterns.

## Results

All 48 study participants had experience working with AMR data from farms in the United States. Thirty-nine of the forty-eight participants were affiliated with academic research institutions, while eight worked for government agencies. Only one participant worked in the private industry as a consultant. We identified four main themes related to on-farm AMR research during the study: (1) navigating access and relationships impacts the quality of the research; (2) despite challenges, more farm-level AMR data is needed because it includes crucial metadata; (3) preserving data integrity requires data sharing protocols; (4) researchers want to transform data into impact.

### Navigating access and relationships impacts the quality of the research

Researchers reported that indicating a desire to share critical additional information that provides context and details about the AMR data (metadata), such as the farm name, location, or antimicrobial use, was usually met with rejection from the farmers. Such reactions stem from farmers’ fear of the repercussions of the data interpretation and their distrust in the ability of the researchers to protect the farm data shared with them.

*“If my research is gonna expose that they have some sort of AMR bacteria, which I’m sure they do because AMR is everywhere. Like, why should they participate?”* (Researcher 26).

Participants also noted farmers’ distrust for government-affiliated individuals or initiatives. Consequently, researchers linked to government agencies reported greater difficulty engaging farmers compared to their counterparts in academic institutions. This sometimes necessitated relying on academic colleagues, which incurred additional costs.

*“… there's a financial cost to us having to work with academia all the time because the costs are so much higher to pay for research through like a university than if we physically went and did it ourselves […]. So it costs the taxpayers a lot of money that we can't go collect it ourselves.”* (Researcher 18)

A farmer’s dissatisfaction with research outcomes, even after the commencement of a project can compromise future research opportunities. For example, discomfort with reported findings could prevent a researcher from carrying out similar studies on other farms, according to one participant.

*“Okay, like again if a farm feels like a researcher has gone too far in identifying them in their publications or research, then It's not only that farmer that you're losing, but those farmers have connections they go to farm conferences they go to a farm options they talk to other farmers and then I know of instances where researchers can no longer enroll farmers because they're not seen as trusting them.”* (Researcher 1)

The approach or actions of different stakeholders in the AMR field, including researchers, also sometimes clash.

*“You know, public health and, let us say veterinary health or animal health, they do not see eye to eye all the time. So many times, you know, If you are a veterinarian or if you work for the USDA, maybe you are trying to protect the viability of a business which is livestock production or poultry production. So you want those organizations to thrive economically. In public health, that’s not what we are working towards. We’re working towards protecting the public’s health. And so if we are trying to look for, you know, is there a risk of certain types of drug-resistant bacteria for example, in the food supply? So I do not ever get access to farm animals… Yeah, so that’s kind of a little just background of my perception of how you know a lot of times it feels like the veterinary health folks are protecting the livestock producers and then public health folks are trying to get access to data but cannot.”* (Researcher 39).

While some stakeholders prioritize protecting the image of the farm and all entities linked to it, other stakeholders support the open release of all forms of data from farms for research. Sometimes these opposing viewpoints make it difficult for a researcher to know how to protect the various data they are entrusted with, especially as different data sources may require different levels of protection.

*“So this is for retail meat collection, we go into the stores, we collect some packages of meat. We record everything we know about that meat, including the brand name and then we do the testing on the meat. For a while we were not releasing the brand name, we thought that that had to be protected. Eventually it turned out that it didn't need to be like we went through a very long process of talking to [US public health agency] lawyers, etc. And now that information is public.”* (Researcher 6)

In addition to the farmers, other stakeholders along the supply chain are concerned about the public’s perception, and so, by extension, they are automatically interested in the image of the farms linked to their name. These stakeholders can sometimes directly influence the decision of the farmers to deny or reject the researchers’ proposals. This is especially true because the farmers do not always own the farms; sometimes they are employed by the corporate bodies to manage them.

*“We ran a study for 3 years and towards the end, the integrator came back and provided pressure against the farmer and said, if you continue to do this study, then we're going to pull out and you're not going to have hogs from us anymore and all that kind of thing, which for them is a livelihood situation and so that farm operator asked us to pull out and to no longer do research there and so we did that and that's just the way it runs sometimes.”* (Researcher 23)

These issues are compounded when individual farms are part of a larger producer hierarchy, requiring multiple layers of approvals.

*“Okay, so there was one time with a company located in the southeastern United States that they we're not allowing me to get onto their farm to conduct some pathogen and AMR related research and so I had to discuss with the farmers. And then discuss with the vertical integration, the next level above the farmers. I had to discuss with them, show them what I was gonna do with the data and then finally, maybe 6 months later, I was allowed to begin the project.”* (Researcher 12)

Delay was a recurring topic. A common example was the delayed release of surveillance or outbreak data by federal agencies. Access to recent surveillance or outbreak data might be difficult because the handlers would withhold the data for years until it lost its relevance.

*“So if you look at other dashboards that federal agencies use, the datasets are typically 3 years behind current time. So I could imagine that 3 years into the future, the AMR data of today would not be that interesting or important. It might be useful for historical purposes in research, but it's not going to be useful for anything else. So I can imagine delay and availability of current data.”* (Researcher 8)

Participants also mentioned experiencing delays while waiting for a farm owner or farm integrator to decide whether to give their consent or not. In many cases, this wait was long enough for researchers to terminate their projects midway and focus on other projects.

*“Well, for us, I would say we tried for probably about 2 years, but we just stopped because we had other pending work that needed to be done.”* (Researcher 6)

The accumulation of these experiences can negatively affect a researcher’s quality of work. Participant 37 described a similar experience where *“it was a little bit frustrating because of course it limited a lot the kind of work you would do because then you would need to have the contacts with the farmers.”* The frustration can often overwhelm researchers and cause them to change their research plans.

*“I think that it would prevent me from asking some questions in the future given how long it took to acquire the data. I might think about if I really want to do that research question in the future because it added 6 months to the project. It's a one year project. It also incurred additional costs that I didn't budget for initially.”* (Researcher 27)

*“They were in business of making money so yeah, the bottom line is that it got published with normal changes and didn't affect the scientific integrity, but I'm not keen to work on those kind of projects again with those kind of companies.”* (Researcher 5)

Although the process to acquire useful livestock AMR data from farmers is often challenging, for some researchers, the negative experiences have not been severe enough to deter them from carrying out their research projects.

*“If everyone at the entire industry said no, I think that I would count that as a barrier. If it's one particular producer, farmer, veterinarian who says no, I won't do it [count it as a barrier] because of this.”* (Researcher 4)

### Despite challenges, more farm-level AMR data is needed because it includes crucial metadata

There was a consensus among participants that researchers do not have access to sufficient and quality AMR data from farms. As previously established, farmers are weary of sharing their farm data due to misinterpretations and negative narratives.

*“So we have 50 farms that have volunteered to have surveillance done. But we really don't have access to any of the other farms, because there's a lot of concern over how that information might be used.”* (Researcher 20)

Some participants were concerned about the possibility of misrepresentation in the available livestock AMR data. Because farm participation is voluntary, there is a good possibility that only a certain demographic of farmers would share their data.

*“…you can never discount that there will be a bias in those farmers giving you consent because they may be A, the ones that are more proactive and hence more open-minded, and hence they will be doing things better. B, the ones that have some kind of a smart view of things and then it will bias their data towards a certain direction. It's not a random sample. So that's a problem”.*

*“So I don't feel like we have great representation in that sense. You only get to sample the farms that are volunteering and therefore you don't have a good distribution.”* (Researcher 20).

Participation bias within any domain can lead to unreliable or completely misleading conclusions, and is of greater concern around sensitive topics like AMR, where it can limit data availability. Although the limited availability of AMR data is a recurring issue, participants highlighted that the unavailability or complete absence of metadata was considered an equally significant challenge for researchers. During the interviews, the term metadata was used to refer to data that gives context to the genotypic and phenotypic AMR results generated from farms. Farmers were reported to be generally apprehensive about providing the metadata because of its potential to be used to identify the farm.

*“Oh, absolutely. They don't want to share their data and later on their story shows up on newspaper, ‘Oh, you know, this is really bad farm and they use so much antibiotics. They have so many AMR bacteria pathogens’ stuff like that, that would be the last thing they need.”* (Researcher 36)

This situation puts researchers in a difficult position. A significant amount of metadata is required to make sense of the AMR data generated from farms. Many participants noted that it is impossible to account for bias without sufficient metadata. Ultimately, they felt that without all the necessary metadata, the AMR data itself could not be accurately interpreted and could lead to unfair consequences for the farmer.

*“So like in antimicrobial usage, if we have just data on which resistance teams are there, but we don't have data on which antibiotics are being newly stored, how they're being used; then we can't really understand those things. And similarly, if we have information on just resistance but not which bacteria was found in, then that's going to be challenging to interpret.* (Researcher 20)

*“Because the epidemiological approach is looking at time and space. So if you de-identify the data in time and space, or certainly in space. Then it's not helpful to do any analysis with. It's just, what's the right way to say.. it's just number counting. It's occurrence, it has no other information. So from a research perspective, it's not very helpful. From a regulatory perspective, it leads to assertions that may or may not be true.”* (Researcher 8)

While researchers who get data directly from farmers say they have better insights about their data, it’s much more challenging for participants who rely on third-party data sources like databases or diagnostic laboratories. When data is uploaded to databases, the authors do not always upload the metadata necessary for contextual analysis. Metadata is also not always prioritized when isolates from farms are submitted to hospitals or laboratories, leaving important holes in diagnostic laboratory databases.

*“But like in our experience, we get cow isolates; we can't even differentiate beef from dairy on a lot of them. I think the age is often recorded. But whether they were treated or not, and with what, I mean, when you start looking at what drugs they are treated with, you won't get enough of that..it's just not captured enough.”* (Researcher 18)

In some cases, the missing metadata can affect more than half of a researcher’s data.

*“And I'm actually in the process of submitting a data release request for the [US public health agency] to see if I can get that because I have like out of 500 isolates I had 300 that I don't know where they were grown from. Is this from a blood sample, poop sample?”* (Researcher 40)

Interestingly, the unavailability of AMR data and its metadata has reportedly been linked to the steps researchers took to protect the farmers and their data. Some participants who have shared their farm-collected AMR data indicated they prefer not to include the metadata to protect the farmers’ trust. Generally, researchers carry a sense of responsibility to preserve a farm’s AMR data whenever they are given consent.

*“And definitely you tell them the data will be de-identified and how you can keep the data anonymous and de-identified. That would be the first thing they need to know. If they know their farm location, names, all other confidential information would be just [be open] to public there's no way they will share the data with you.”* (Researcher 36)

In an attempt to identify the core issue contributing to paucity of quality AMR data and metadata, one participant hinted that a lack of clear, standardized rules for data sharing was responsible for the dissatisfaction researchers experienced.

*“We don't have like you know say we set protocol or you have to do you know do this I don't think right now there is this kind of set protocol. So we go by our intuition, that's it.”* (Researcher 17)

### Preserving data integrity requires data sharing protocols

Although there are no standard procedures for AMR data sharing, some researchers have developed their protocols for safe and secure farm-level data sharing,

*“…we already have sort of policies and procedures in place for data privacy. And so we typically...AMR gets rolled into those protections.*” (Researcher 13)

The most prominent security measure adopted by researchers when sharing their farm-level AMR data is to anonymize it, primarily through its metadata. They also remove some granular details and replace them with aggregated levels of the data.

*“Okay, yeah, so I'd say generally we share data. The metadata is shared. The thing that is sort of restricted is typically the names of submitters of farmers, of veterinarians, and then the locations are typically restricted to things like zip codes. We don't have specific addresses, you know, it's more of a region associated with that. So those are the... any sort of personal identifiable information along with locations that might be identifiable are typically not used as part of the data sharing.”* (Researcher 13)

Other researchers/agencies rely on special tools or secure servers coupled with encryption or multi-authentication requirements to share AMR among colleagues or team members securely. And in some cases, those servers are managed by professional experts to ensure efficacy.

*“I mean we keep it on a secure server. Access to that server is provided by our IT people, with my permission. And then they have to know something about like what samples are what? And so there's like a decoding of that, Cause we have lots of different sequence data in our lab. And so they would need to be kind of on that project to kind of get to the data.”* (Researcher 41)

Non-disclosure agreements have also been used by researchers, farmers, and even research institutions to secure livestock AMR data at the farm level.

*“I'm trying to think of like any other processes that we've had to go through for other types of data sharing. In some cases we've got to sign NDAs or things like that [...]”* (Researcher 19)

Some researchers do not stop at the sharing stage; they go all the way to ensure data privacy is prioritized by the colleagues with whom they share their data.

*“And we try in the research that we've done in house with our collaborators, we try to always stay involved in the interpretation of the data. So that somebody who is not familiar with why these isolates were grown or how antibiotic treatment works in agricultural settings and so on. They gonna write a paper or conclusion that don't really make sense or could be misinterpreted. So we've been trying to be monitoring what is it, this research that we do if we are working with collaborators. What is it that our collaborators are writing and if we think that it makes sense what they are writing based on the data and the results that they are finding and giving that perspective of "okay, this makes sense this is where the data is coming from or not.”* (Researcher 31)

Overall, antimicrobial resistance data sharing is considered a sensitive issue due to its potential adverse consequences for individuals, businesses, or even entire countries and regions.

*“If you just focus on the research use, it's fine. But the important thing is non-researchers will use it to make much bigger decisions than researchers will, and that could have some tremendous negative consequences that are outweigh the benefits of research. Because we will indirectly reward countries and regions that don't do monitoring, right? Because it will look like they don't have a problem.”* (Researcher 38)

### Researchers want to transform data into impact

Researchers may find themselves unable to participate in good faith in the policy development work their research inspires due to lack of detail in their data limiting their analytical methods.

*“I mean, maybe just knowing trends and antimicrobial susceptibility and the resistance genes associated with it. Outside of that, it would be the only thing I would find useful.”* (Researcher 3)

Most of the participants interviewed for this study used farm-level antimicrobial resistance data to carry out surveillance and risk analysis research. They were generally interested in studying the distribution of antimicrobial resistance.

*“… for us, you know, we just basically want to see you know, the bacteria that we're getting, what the resistance there is, and is there differences between locations.”* (Researcher 10)

For others, it was valuable to use the antimicrobial resistance data generated on the farms to make clinical decisions.

*“We use this for diagnostic purposes mostly things like antibiograms.”* (Researcher 13).

These simple analyses may not feel sufficient to researchers trying to guide policy to address the spread of AMR.

*“Maybe a salmonella that we find and publish the sequence of, somebody will identify as a problem and point to us as a source and then policies may change, but I don't think that's gonna happen.”* (Researcher 4)

Due to the complexity of antimicrobial resistance, a lot is still unknown.

*“So our major research questions are. How do antibiotics change the assembly and diversity of microbial communities? How various either antibiotic use or other management would change the abundance of resistance genes, trying to identify risk factors for increasing antibiotic resistance. We also want to know if resistance genes can be horizontally transferred from one bacterium to another, so we look for markers of, we call the mobile genetic elements so those are like transposons and plasmids that can give resistance genes to other bacteria. Yeah, those are the big questions.”* (Researcher 25)

For some researchers, studying the evolution of mechanisms and forms of antimicrobial resistance is an important step toward understanding AMR.

*“Well, we get an isolate, which is, you know, bacterium. We sequence it, so we collect DNA, we sequence it, we get the genomic data. And then we will do different analysis looking at presence-absence of antimicrobial resistance genes, looking at how related they are, building sort of family trees, phylogenetic trees. And then using those often along with data on location to see how organisms spread across the US or across the world. And whether they have adapted to specific animal species? Let's say they're only found in cows and rarely found in humans. Or if found cows and chickens and pigs and everything else, which will help us to better understand how these organisms emerged, where it came from, and how likely there are to be human health issues…but also how they [antimicrobial resistant organisms] evolve, how they change over time.”* (Researcher 38)

Others focus on examining the relationship between phenotypic and genotypic resistance to predict antimicrobial resistance patterns. Some researchers also employ machine learning algorithms to improve prediction accuracy.

*“We use broth micro dilution for that, we do whole genome sequencing on isolates of specific bacteria, and then do resistance gene profiling, and then predict antibiotic resistance phenotype based on the genotype”* (Researcher 2)

Some researchers were interested in investigating the risk animals pose to humans and vice versa. Overall, the One Health concept that involves investigating the transmission of resistance among humans, animals, and the environment emerged as a central focus for researchers that use antimicrobial resistance data.

*“So we are looking at the overall impact of agriculture on AMR, looking at it from a one health perspective, of course we are focused on the environmental side of things. So a cyclical effect.”* (Researcher 12).

The spillover of antimicrobial resistance from wildlife to farms is also considered a priority for antimicrobial resistance transmission research.

*“We did a spatial analysis of where feral pigs were residing near domestic pigs raised outside and that's where we sampled domestic pigs. So we sampled and then brought samples to the lab and looked for E. coli and then and all the positive samples were sent to our genomics lab on campus for whole genome sequencing.”* (Researcher 1)

One participant mentioned harnessing the potential of antimicrobial resistance data for educating farmers.

*“We basically are kind of trying to use that information to develop some materials that can educate the farmers that we work with.... Because you know all of these informations we discussed with our stakeholders when we have some results. What we find and what we do not find. So in addition to the food-borne pathogens, so we also discuss up some of these issues.”* (Researcher 43)

All of this basic research is a necessary precursor to science-based policy around AMR in livestock. However, access to farms and farm-level data is necessary to develop these truly impactful research programs.

## Discussion

The present study is designed to identify the experiences researchers encounter when conducting farm AMR research in the United States, the decisions they make to protect and utilize the farm data, and their research goals. We found that research quality is impacted by access to farms and to farm-level metadata, and that protocols are needed to ensure that access preserves farm privacy.

For many researchers, getting the cooperation of farmers to contribute data for AMR research felt like an uphill battle. This contradicts findings that farmers in the United States were very enthusiastic about supporting research projects on the farms and wanted to volunteer their resources for research (18). A possible reason for this could be that, unlike other farm-related research, the risks of AMR research for farmers can sometimes outweigh its benefits, therefore swaying farmers’ inclination to support its research. Due to the concentrated geographical distribution of farms in the US, farms involved in research studies can often be traced even when location data is aggregated at the state level (19). Consequently, farmers may face severe socioeconomic repercussions, especially when a public health concern like AMR is associated with their farm (7). These incidents can lead to public harassment, farm product recalls, and sometimes complete business closure. In one case, the fallout resulted in a daily sales loss of $1.8 million in Saudi Arabia alone, affecting more than 9,000 farmers (20). We suggest that events like this motivate farmers’ reluctance to support researchers conducting antimicrobial resistance research. Our assumption aligns with Zhang et al.’s (21) theory that the willingness of a farmer to share their farm data is not only associated with the stakeholder requesting the data but also the risk that the farm data poses. Our study also found that researchers experienced similar farmers’ reluctance regardless of the data protection technique they proposed to use, which aligns with the finding that farmers expressed fear of being penalized or punished for their data, despite the removal of all identifiable information from the farm data collected (22).

The power dynamics among other stakeholders along the supply chain are another factor that significantly influenced researchers’ experiences by hindering access to farm data. The influence of stakeholders within the supply chain network on farmers’ decision-making processes has garnered significant attention in recent years (8, 23). Farmers reportedly do not have autonomous power to make decisions in the agricultural food production system, and they often have to share the decision-making power with other stakeholders in the supply chain (23). This is because, in some countries, a few corporations control almost all agribusiness activities, including food production, processing, and distribution. In addition to the fact that the integrators often own the livestock, the farmers are also given contracts detailing the production techniques and routines to be used on the farm, even the type of feed that the animals consume (24). As a result of this system, the desires of the integrators can sometimes be misaligned with those of the farmers running the farm; hence, when researchers successfully convince farmers about the need for AMR research data, they may also have to convince the other stakeholders along the supply chain.

The complex management network within the livestock production system may not just delay researcher access, but it can also affect the quality of the data that researchers eventually get access to. As many participants in this study reported, the quality of AMR farm data and metadata leaves much to be desired. Relevant data, such as the class of antimicrobials used on the farm, the antimicrobial use rate, and disease outbreaks, are largely unavailable to researchers. Global organizations like the World Organisation for Animal Health recognize this need, hence why one of its key objectives in its global action plan focuses on making animal AMR data from different countries more accessible in real-time. These types of data provide necessary context to the phenotypic and genotypic AMR data, directly influencing the depth and scope of research conducted in the field. As Schnall et al. (25) explain, the unavailability of sensitive yet crucial data forces an immense dependence on modeling estimates and limits the reliability of scientific conclusions.

While this study accurately documents the encounters of researchers conducting AMR research on farms in the United States, it is essential to note that these experiences may not apply to all researchers in every region of the world. For example, the unavailability of quality data observed in Africa or South America could be associated with insufficient funding and the underdeveloped capacity of research institutions in those regions (26, 27). Therefore, there is a need to investigate other forms of social barriers that are peculiar to other regions, outside of the United States (28). Furthermore, there is an urgent need to develop AMR data sharing systems that protect farmers and other stakeholders in livestock production, while simultaneously fostering innovation and advancing research in antimicrobial resistance.

## Conclusion

Considering the significant role that livestock farms can play in helping us understand the novel mechanisms of AMR, it is essential to conduct impactful AMR research in this sector. To support that research, there is an urgent need to close the gaps in data quality, accessibility, and availability. To that end, protocols and best practices are needed for research access to AMR data and metadata on and around livestock facilities, supporting necessary research while protecting farms.

## Data Availability

The raw data supporting the conclusions of this article will be made available by the authors, without undue reservation.
